# Quality Evaluation of Decoction Pieces of *Fagopyri Dibotryis Rhizoma* Based on HPLC Fingerprint, Q-TOF-MS/MS, and Chemical Pattern Recognition Qualitative Analysis Combined With Multicomponent Quantitative Analysis

**DOI:** 10.1155/jamc/2328049

**Published:** 2025-11-17

**Authors:** Yi Cao, Meng He, Yaru Feng, Yujia Li, Nan Qin, Zhihua Dou

**Affiliations:** ^1^School of Pharmacy, Nantong University, Nantong 226019, Jiangsu, China; ^2^Zhangjiagang Zhonghui Medical Plastic Technology Co., Ltd., Suzhou 215600, Jiangsu, China; ^3^Department of Pharmacy, Affiliated Nantong Hospital 3 of Nantong University, Nantong 226006, Jiangsu, China; ^4^Department of Pharmacy, Nantong Third People's Hospital, Nantong 226006, Jiangsu, China; ^5^Department of Pharmacy, Nantong Hospital of Traditional Chinese Medicine, Nantong 226001, Jiangsu, China

## Abstract

*Fagopyri Dibotryis Rhizoma* (FDR), the dried rhizome of *Fagopyrum dibotrys* (D. Don) Hara (*F. dibotrys*), is a famous herbal drug with a long application history in China. Recently, FDR and its preparations have attracted wide attention due to their therapeutic values for coronavirus disease 2019 (COVID-19) and COVID-19-related acute lung injury. There is a significant difference in quality among FDRs from different habitats, which can seriously affect the clinical efficacy. The original medicinal materials can only be used in the clinic after being processed into decoction pieces, but there is currently a lack of comprehensive quality evaluation of FDR decoction pieces prepared using FDRs from different origins. In this study, the HPLC fingerprints of 23 batches of FDR decoction pieces prepared using FDRs from 8 provinces such as Yunnan and Guizhou in China were established; 47 common peaks in these fingerprints were marked, among which, 80 components were identified by Q-TOF-MS/MS, including 32 tannins, 17 phenols, 12 flavonoids, 11 phenylpropanoid glycosides, 3 amino acids, 2 organic acids, 1 terpenoid, 1 alkaloid, and 1 other component; the chemical pattern recognition analysis, including hierarchical cluster analysis, principal component analysis, and orthogonal partial least squares discriminant analysis, was conducted by using the quantified peak areas of the common peaks as variables; and the contents of 4 tannins such as procyanidin B1, procyanidin B3, procyanidin C1, and procyanidin A2, 3 phenolics such as gallic acid, protocatechuic acid, and protocatechualdehyde, and 3 flavonoids such as catechin, epicatechin, and epicatechin gallate were determined in 23 batches of FDR decoction pieces. The results indicate that there is a significant difference in the quality between the decoction pieces prepared using FDRs from Yunnan and Guizhou and those prepared using FDRs from 6 other provinces, and the former have a better quality compared with the latter.

## 1. Introduction

*Fagopyri Dibotryis Rhizoma* (FDR), the dried rhizome of *Fagopyrum dibotrys* (D. Don) Hara (*F. dibotrys*) belonging to the family *Polygonaceae* and genus *Fagopyrum* [1, 2], is a famous herbal drug with a long application history in China [3, 4]. Some experimental results indicate that FDR has a variety of pharmacological activities, such as antioxidant, anti-inflammatory, antibacterial, antiviral, anticancer, antidiabetes, and immunoregulation activities [3, 4]. In clinical practice, FDR and its preparations, namely, FDR capsules, FDR tablets, and Wei Mai Ning capsules have been used to treat many lung diseases such as lung abscess and lung cancer and intestinal diseases such as ulcerative colitis [3–5]. Recently, FDR and its preparations have attracted wide attention due to their therapeutic values for coronavirus disease 2019 (COVID-19) and COVID-19-related acute lung injury [6–8]. Zhang et al. used the computer model to screen 26 Chinese herbal medicines with the most components that can directly inhibit COVID-19 virus, and FDR is one of these medicines [9].

**Figure 1:**
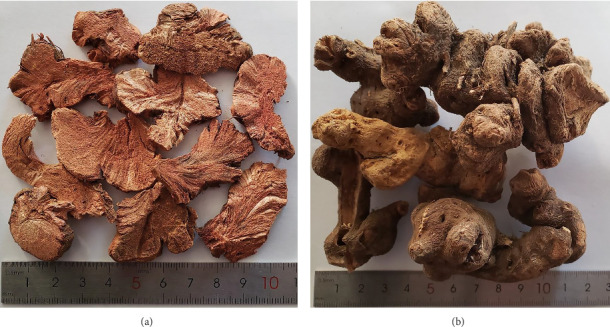
Decoction pieces of FDR (a) and original medicinal materials of FDR (b).

*F. dibotrys* is native to southwest China and is mainly distributed in the southern region of the Yellow River (97°–121° E; 21°–32° N), including Yunnan, Guizhou, Sichuan, Anhui, Jiangsu, Hubei, Henan, and Shaanxi [10]. The components contained in FDR include tannins, phenolics, flavonoids, phenylpropanoid glycosides (PGs), organic acids, terpenoids, and amino acids [3–6, 11, 12], of which, the tannins, flavonoids, and phenolics are considered to be the main active components of FDR [3, 4]. The contents of the components in *F. dibotrys* grown in different ecological environments vary greatly, resulting in the uneven quality of FDRs from different origins in the market, which seriously affect the clinical efficacy [10]. Wu et al. determined the contents of 24 components in FDRs from Yunnan, Guizhou, Sichuan, Hubei, and Jiangsu provinces in China and conducted chemical pattern recognition analysis using the contents of these 24 components as variables. The results showed that there is a significant difference in quality among FDRs from different habitats, and the quality of FDR from Yunnan is most superior [13]. However, the original medicinal materials can only be used in clinical practice after being processed into decoction pieces [14]. Decoction pieces of FDR (Figure 1(a)) are the products of the original medicinal materials of FDRs (Figure 1(b)) after being washed, moistened, cut into thick pieces, and dried [1]. To the best of our knowledge, there is currently no literature on the comprehensive quality evaluation of FDR decoction pieces prepared using FDRs from different origins. Our research team has previously established a method for comprehensively evaluating the quality of herbal drugs based on HPLC fingerprint, Q-TOF-MS/MS, and chemical pattern recognition qualitative analysis combined with multicomponent quantitative analysis [15]. In this study, we used this method to evaluate the qualities of 23 batches of FDR decoction pieces prepared using FDRs from Yunnan, Guizhou, Sichuan, Jiangsu, Anhui, Hubei, Henan, and Shaanxi provinces in China, aiming to provide a reference for rational drug use in clinical practice.

## 2. Experimental

### 2.1. Chemicals and Reagents

Reference substances gallic acid (purity ≥ 91.5%), protocatechuic acid (purity ≥ 97.5%), protocatechualdehyde (purity ≥ 99.9%), epicatechin gallate (purity ≥ 98.1%), and rutin (purity ≥ 91.6%) were purchased from the National Institutes for Food and Drug Control (Beijing, China). Procyanidin B1 (purity 98.0%), procyanidin B3 (purity 98.0%), catechin (purity 98.0%), procyanidin B2 (purity 98.0%), epicatechin (purity 98.0%), procyanidin C1 (purity 98.0%), and procyanidin A2 (purity 98.0%) were purchased from Nanjing Guangrun Biological Products Co., Ltd. (Nanjing, China). Procyanidin C2 (purity ≥ 98.9%) was purchased from Sichuan Weikeqi Biotechnology Co., Ltd. (Chengdu, China). LC/MS-grade acetonitrile and HPLC-grade methanol were provided by Fisher Scientific (Fair Lawn, NJ, USA). HPLC-grade formic acid was purchased from Sinopharm Chemical Reagent Co., Ltd. (Shanghai, China). Purified water was provided by Wahaha Group Co., Ltd. (Hangzhou, China).

### 2.2. Samples and Sample Solutions Preparation

A total of 23 batches of FDR decoction pieces were purchased from the production enterprises of herbal pieces, and the information of 23 samples is shown in Table 1.

FDR decoction pieces were pulverized before use. Two g of sample powder was weighted and put into a 50 mL volumetric flask, 50 mL of 50% (v/v) ethanol was added to the mark of the volumetric flask, and the mixture was extracted (53 kHz, 200 W) in ultrasonic cleaner for 30 min and finally ultrasonicated (53 kHz, 200 W) for 30 min. After being cooled, the extracts were filtered and the filtrates were concentrated under reduced pressure at 50–70°C until near dryness. The residues were washed with 10% (v/v) acetonitrile in batches. The washing solution was then put into a 10-mL volumetric flask and diluted to the mark with 10% (v/v) acetonitrile. The mixture was centrifuged at 3000 rpm for 5 min after being shaken well. The supernatant was collected, filtered with a 0.22-μm microporous membrane, and the resulting filtrate was used as the sample solution.

### 2.3. Reference Substance Solutions Preparation

An appropriate dose of each reference substance mentioned in Section 2.1 was weighed precisely and put into the same volumetric flask, dissolved, and diluted with 50% (v/v) ethanol, and the mixed reference substance solution containing 13 components with a concentration of about 40 μg/mL was prepared for identifying the common peaks.

Appropriate doses of 10 reference substances were weighed precisely, dissolved, and diluted with 50% (v/v) ethanol to make 10 stock solutions of gallic acid, protocatechuic acid, protocatechualdehyde, procyanidin B1, procyanidin B3, catechin, epicatechin, procyanidin C1, epicatechin gallate, and procyanidin A2, respectively.

Appropriate volumes of the stock solutions of 10 reference substances were precisely taken and mixed, and then diluted with 50% (v/v) ethanol to make the working solution A for content determination; the concentrations of above 10 reference substances were 102.025, 86.275, 12.525, 300.4, 315.6, 277.48, 397.28, 1047, 427.92, and 618 μg/mL, respectively. 50% (v/v) ethanol was used to dilute the working solution A by 2, 5, 10, 20, and 50 times to make the working solutions B–F, respectively.

### 2.4. Detection of HPLC Fingerprint and the Contents of 10 Components

The HPLC fingerprints of 23 batches of FDR decoction pieces and the contents of 10 components, including 4 tannins such as procyanidin B1, procyanidin B3, procyanidin C1, and procyanidin A2, 3 phenolics such as gallic acid, protocatechuic acid, and protocatechualdehyde, and 3 flavonoids such as catechin, epicatechin, and epicatechin gallate, were detected in 23 samples using a Waters Alliance HPLC system (consisting of an e2695 separation unit, a 2998 PDA detector, and an Empower 3 data processing system, Waters Corp., USA) and a Symmetry C_18_ column (4.6× 250 mm, 5 μm, Waters Corp., USA). The injection volume was 30 μL. The mobile phase consisted of acetonitrile (A) and 0.1% (v/v) formic acid (B), and the gradient program was as follows: 0–10 min, 2%–4% A; 10–20 min, 4% A; 20–30 min, 4%–10% A; 30–50 min, 10%–12% A; 50–60 min, 12%–15% A; 60–70 min, 15%–16% A; 70–100 min, and 16%–50% A. The flow rate was set at 1.0 mL/min, the column temperature was maintained at 30°C, and the detection wavelength was 280 nm.

### 2.5. Validating the HPLC Method for Fingerprint Analysis

The precision, stability, and repeatability tests were performed to validate the HPLC method for detecting fingerprints, in which, peak 25 (procyanidin C1) was taken as the reference peak, and the relative standard deviation (RSD) of relative peak area (RPA) and the mean relative retention time (RRT) of 21 common peaks were measured. Sample solution 20 was injected 6 times continuously within a day for precision assessment and was then injected, respectively, at 0, 6, 9, 12, 18, and 36 h after being prepared for stability assessment. Six sample solutions were made from S20 in parallel for repeatability assessment.

### 2.6. Establishing HPLC Fingerprints and Identifying Common Peaks

The chromatographic data of 23 batches of FDR decoction pieces were imported into the Similarity Evaluation System for Chromatographic Fingerprint of TCM (2012 edition, Chinese Pharmacopoeia Commission, China). The chromatogram of S23 was taken as a reference chromatogram, and common peaks in this reference chromatogram were marked.

The components of common peaks were identified using an UFLC-Q-TOF-MS/MS (consisting of an SIL-20AC XR autosampler, a LC-20AD XR quaternary pump, and an SPD-M20A DAD detector, Shimadzu, Japan) system. The same chromatographic column, mobile phases, and gradient conditions as described in Section 2.4 were used to separate the components and then a Triple TOF 4600 system (AB Sciex, USA) was used for obtaining mass spectra in negative ion mode with the DuoSpray Ion source. The mass spectrometric parameters were set as follows: ion spray voltage floating (ISVF) 4500 V, source temperature (TEM) 500°C, curtain gas (CUR) 35 psi, nebulizer gas (Gas 1) 60 psi, and heater gas (Gas 2) 60 psi. The mass spectrometry data were obtained using the TOFMS-IDA-10MS/MS method based on the following relevant parameters: collision energy (CE) −10 eV; decluster potential (DP) −80 V, accumulation time 250 ms, mass range for detecting TOF-MS 115–1500 Da, CE −35 eV, collision energy spread (CES) 15 eV, DP −80 eV, accumulation time 100 ms, and mass range for detecting TOF-MS/MS 50–1500 Da. PeakView 1.6 mass spectrometry analysis software (AB Sciex, USA) was used for analyzing the LC–MS/MS data.

### 2.7. Chemical Pattern Recognition Analysis

The precise amounts of samples in the injected solution were used to quantify the peak areas of 47 common peaks in HPLC fingerprints and then the quantified peak areas were taken as variables to perform hierarchical cluster analysis (HCA), principal component analysis (PCA), and orthogonal partial least squares discriminant analysis (OPLS-DA).

### 2.8. Validating the Method for Content Determination

The method for content determination was validated through detecting the linear relationship, limit of detection (LOD), limit of quantitation (LOQ), precision, stability, repeatability, and recovery of the 10 components mentioned in Section 2.4, respectively. Accurately 30-μL solution from each of working solutions A, B, C, D, E, and F was injected, respectively, into the HPLC system to calculate the calibration curves, correlation coefficients, and linear ranges of 10 components to detect linear relationship. Various concentrations of the reference substance solutions were prepared by successive dilutions of working solution F with 50% (v/v) ethanol. The signal-to-noise ratios of 3:1 and 10:1 were used, and 10-μL solution from each of various concentrations of reference substance solutions was injected, respectively, to determine the values of LOQ and LOD. The RSDs of the peak areas of the 10 components were measured to perform intraday and interday precision tests and sample stability test, respectively. The intraday precision was detected by 6 consecutive injections of 30-μL working solution C, and the interday precision was detected by injections of 30-μL working solution C twice daily for 3 consecutive days. The peak areas of 10 components detected in stability test in Section 2.5 were analyzed for stability assessment. The concentrations of 10 components were calculated based on the peak areas of these 10 components measured in repeatability test in Section 2.5, and the RSDs of the concentrations were detected for repeatability assessment. The recovery test involved precise weighing of 1 g of S20 powder and addition of 10 reference substance stock solutions prepared in Section 2.3 to the sample with a specific volume based on the approximate ratio of sample content to reference substance (1:1), and 6 sample solutions were prepared in parallel. These 6 sample solutions were injected into the HPLC system to calculate the mean recovery rates and RSDs of 10 components.

The methods employed in this study were in line with those proposed by the authors of reference literatures [14, 15].

## 3. Results and Discussion

### 3.1. Validating the Method for HPLC Fingerprint Analysis

The RSDs of RPA and RRT for precision were below 4.48% and 0.33%, respectively, those for stability were not greater than 4.97% and 0.44%, respectively, and those for repeatability were **≤** 4.96% and 0.72%, respectively. This finding conformed to national standards for TCM fingerprinting [14, 15].

### 3.2. Establishing the HPLC Fingerprints and Identifying the Common Peaks

**Figure 2:**
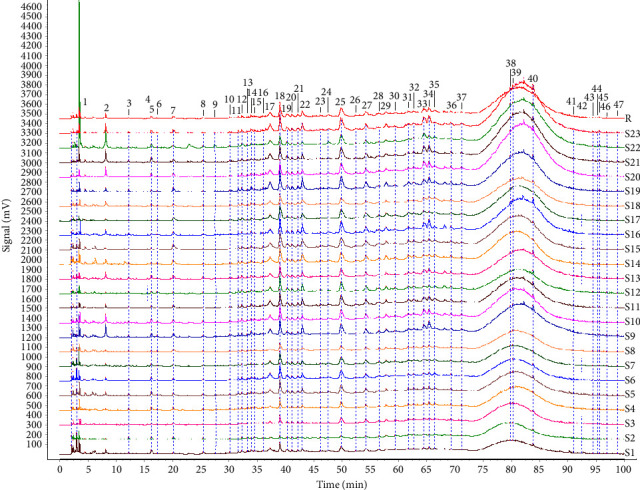
Chromatograms of the reference chromatogram (R) and 23 batches of FDR decoction pieces (S1–S23).

**Figure 3:**
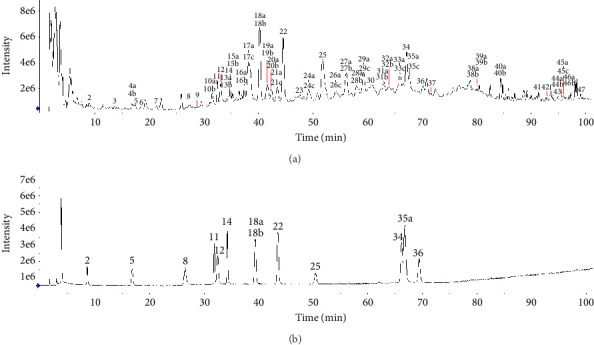
Total ion chromatograms of sample (a) and mixed reference substances (b) (negative ion mode).

**Figure 4:**
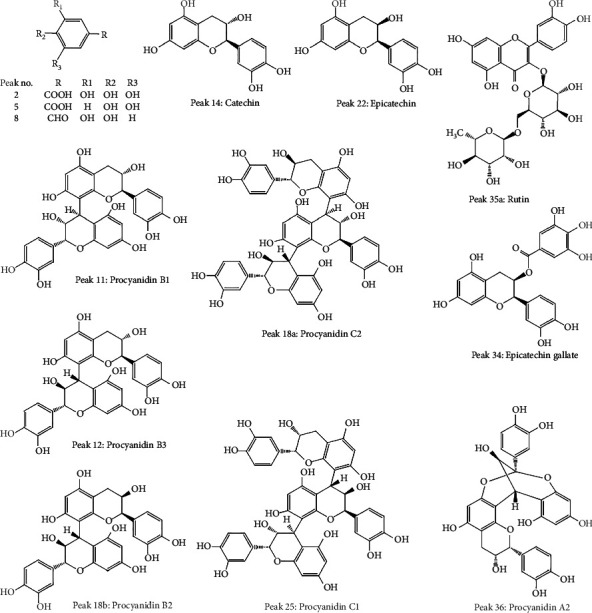
Structures of the 13 components confirmed by comparison with reference substances.

The chromatograms in Figure 2 showed that 47 common peaks were marked in the HPLC fingerprint of 23 batches of FDR decoction pieces. The identification process of the components of the common peaks was as follows. First, the total ion chromatograms of the samples and mixed reference substances (Figure 3) were extracted by a mass spectrometry analysis software (PeakView 1.6). Second, the analysis of the mass spectral data of the reference substances and the summarization of the dissociative rules of the reference substances revealed that the quasimolecular ion [M-H]^−^ or [M+Cl]^−^ or 1/2[M-2H]^2−^ could be selected as the precursor ion to produce MS/MS product ions. Finally, the components of the 47 common peaks were identified by comparison of the retention time, m/z of [M-H]^−^ or [M+Cl]^−^ or 1/2[M-2H]^2−^, and MS/MS fragmentation patterns with those of the reference substances or previous literature reports, in combination with online retrieval of the compound database of PubChem (https://pubchem.ncbi.nlm.nih.gov). As shown in Table 2 and Figure 3, 80 components were identified in 47 common peaks of the HPLC fingerprints of FDR decoction pieces, including 32 tannins, 17 phenolics, 12 flavonoids, 11 PGs, 3 amino acids, 2 organic acids, 1 terpenoid, 1 alkaloid, and 1 other component. Among them, 13 components were confirmed by comparison of the mass spectrometry data of them with those of the reference substances, containing 6 tannins such as procyanidin B1, procyanidin B3, procyanidin C2, procyanidin B2, procyanidin C1, and procyanidin A2, 3 phenolics such as gallic acid, protocatechuic acid, and protocatechualdehyde, 4 flavonoids such as catechin, epicatechin, epicatechin gallate, and rutin, which were shown as Peaks 11, 12, 18a, 18b, 25, 36, 2, 5, 8, 14, 22, 34, and 35a, respectively. The structures of these 13 components were depicted in Figure 4.

### 3.3. Chemical Pattern Recognition Analysis

The quantified peak areas of 47 common peaks of 23 batches of samples were used as variables to perform HCA, PCA, and OPLS-DA.

**Figure 5:**
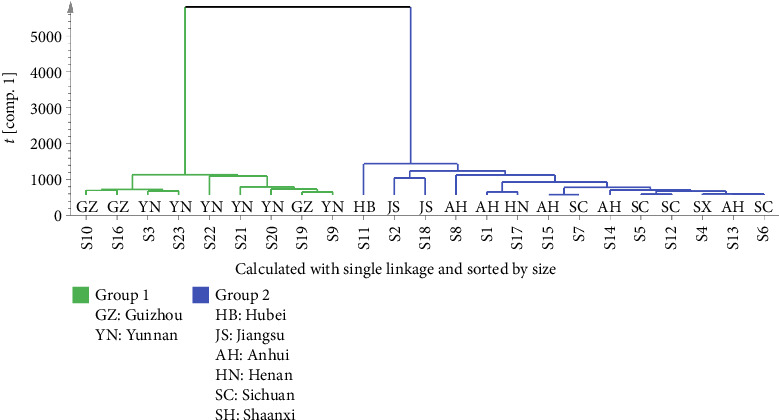
HCA results of 23 batches of FDR decoction pieces.

HCA is an unsupervised pattern recognition method, which is commonly used to classify samples into different groups based on the characteristics of variables and determine the degree of similarity between the samples [15, 20]. In the present study, the quantified peak areas of 47 common peaks of 23 batches of FDR decoction pieces were introduced into SIMCA14.1 (Umetrics, Sweden) to perform HCA. The results (Figure 5) show that 23 batches of FDR decoction pieces were classified into two groups: S3, S9, S10, S16, and S19–S23 (FDRs from Yunnan and Guizhou province) were clustered into one group, S1, S2, S4–S8, S11–S15, S17, and S18 (FDRs from 6 other provinces) were clustered into another group; it was indicated that there was a significant difference in quality between FDR decoction pieces prepared using FDRs from Yunnan and Guizhou provinces and those prepared using FDRs from 6 other provinces.

**Figure 6:**
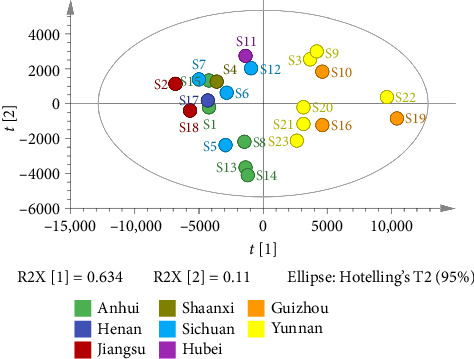
PCA score plot of 23 batches of FDR decoction pieces.

The unsupervised pattern recognition PCA does not require prior knowledge of the datasets, which effectively amplifies the difference and facilitates the sorting of factors [15]. The PCA score plot was utilized for the purpose of detecting the difference among the samples [15, 21]. In this study, the quantified peak areas of 47 common peaks of 23 batches of FDR decoction pieces were introduced into SIMCA14.1 to generate the score plot in Figure 6. As presented in Figure 6, the first two principal components (PCs) explained 74.4% (PC1, 63.4% and PC2, 11.0%) of the total variation, and S3, S9, S10, S16, and S19–S23 (FDRs from Yunnan and Guizhou province) could be distinguished clearly from S1, S2, S4–S8, S11–S15, S17, and S18 (FDRs from 6 other provinces). This finding was consistent with that of HCA, suggesting that there was an obvious difference in quality between FDR decoction pieces prepared using FDRs from Yunnan and Guizhou provinces and those prepared using FDRs from 6 other provinces.

**Figure 7:**
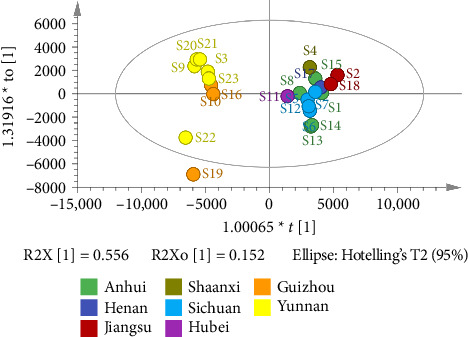
OPLS-DA score plot of 23 batches of FDR decoction pieces.

**Figure 8:**
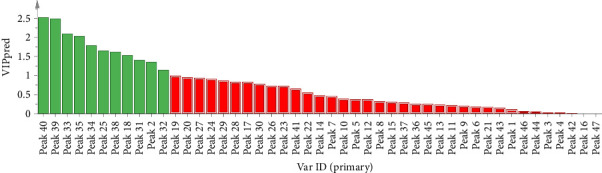
VIP plot of 47 common peaks.

**Figure 9:**
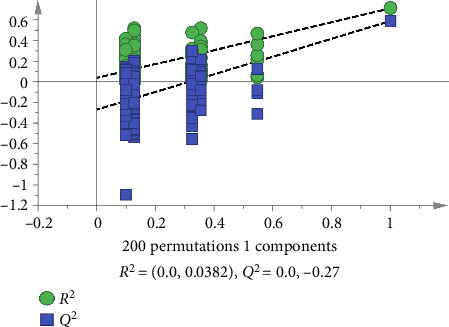
Permutation test results of the OPLS-DA model (*n* = 200).

The OPLS-DA model for supervised chemical pattern recognition is designed to optimize classification and identify the potential markers of variation in samples [15]. In this study, the OPLS-DA model was established by SIMCA14.1 software using the quantified peak areas of 47 common peaks of 23 batches of FDR decoction pieces as variables. The values of *R*_*X*cum_^2^, *R*_*Y*cum_^2^, and *Q*_cum_^2^ were 0.884, 0.376, and 0.191 accordingly, suggesting that the abovementioned model had good fitting and predictive ability [15]. The score plot in Figure 7 revealed that S3, S9, S10, S16, and S19–S23 (FDRs from Yunnan and Guizhou provinces) and S1, S2, S4–S8, S11–S15, S17, and S18 (FDRs from 6 other provinces) were located in two different regions; the findings were consistent with those of the HCA and PCA. The contribution of the variable to group classification was assessed using the variable importance in the projection (VIP). A higher VIP indicated that the variable had a greater effect on classification. Variables with VIP > 1 was mainly used for sample discrimination [15]. VIP plot in Figure 8 revealed that 11 common peaks with VIP > 1 were identified, including peak 40 (6-acetyl-3′,6′-di-p-coumaroylsucrose and N-trans-feruloyltramine), 39 (procyanidin B4-3′-O-gallate and taxifolin 3-O-β-D-xyloside), 33 (ellagic acid, 3,3′-digalloylprocyanidin B2, and arecatannin A1 3,3′-digallate), 35 (rutin, isocinnamtannin A2, and catechin-(4β ⟶ 8)-catechin-(4β ⟶ 8)-epicatechin), 34 (epicatechin gallate), 25 (procyanidin C1), 38 (tricin 4′-O-(erythro-β-guaiacylglyceryl) ether 7-O-glucoside and 1-p-coumaroyl-6-feruloyl-sucrose), **18** (procyanidin C2 and procyanidin B2), 31 (taxifolin-3-glucoside and isolariciresinol 9′-O-glucoside), 2 (gallic acid), and 32 (epicatechin 5-glucoside and taxifolin), indicating that these 21 components were the markers for discriminating between FDR decoction pieces prepared using FDRs from Yunnan and Guizhou provinces and those prepared using FDRs from 6 other provinces. A total of 200 permutation tests were conducted to determine the validity of the OPLS-DA model, and the vertical intercept values of *R*^2^ and *Q*^2^ were 0.0382 and −0.27, respectively (Figure 9); this indicated that the established model addressed the issue of overfitting and had a good predictive effect [15, 20].

### 3.4. Validating Method for Content Determination

Table 3 shows that a higher correlation coefficient value (*R*^2^ > 0.9998) exhibited excellent linearity over a broad range of injected amounts.  Table 4 shows that all RSDs of the intraday and interday precision, stability, and repeatability were below 5%, the mean recovery rate ranged from 97.36% to 101.76%, and RSD ranged from 0.75% to 1.89%. The abovementioned findings satisfied the requirements of analytical method for drug quality standard specified in Chinese Pharmacopoeia [1].

### 3.5. Content Determination of 10 Components in 23 Batches of FDR Decoction Pieces

**Figure 10:**
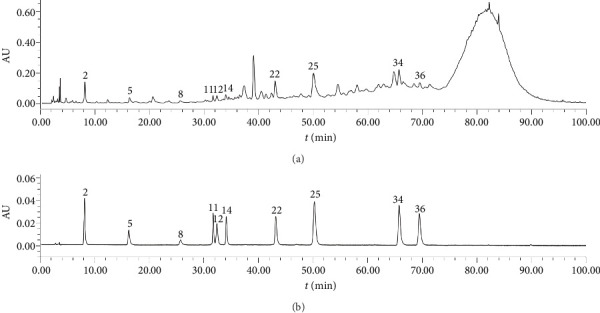
HPLC chromatograms of sample (a) and mixed reference substances (b). The number of peaks was the same as in Table 2.

**Figure 11:**
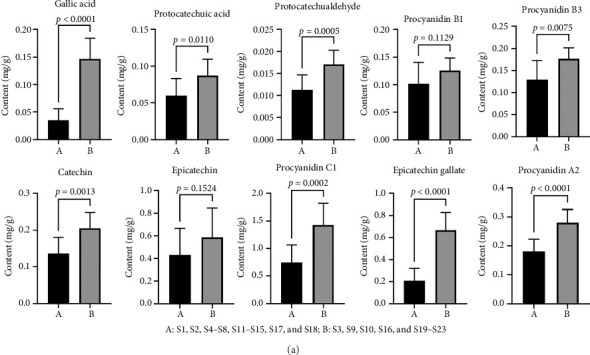
Bar plots of contents of the 10 components in 23 batches of FDR decoction pieces.

The chromatograms of the samples and mixed reference substances are shown in Figure 10, and the content determination results of 10 components in 23 batches of FDR decoction pieces are shown in Table 5. Table 5 revealed that there existed differences in the contents of 10 components among different samples. GraphPad Prism 9 (GraphPad Software Inc. USA) was applied to comparatively analyze the contents of 10 components between 9 batches of FDR decoction pieces (S3, S9, S10, S16, and S19–S23) and 14 batches of FDR decoction pieces (S1, S2, S4–S8, S11–S15, S17, and S18).  Figure 11 shows that, except for procyanidin B1 and epicatechin, the average contents of other 8 components in S3, S9, S10, S16, and S19–S23 were significantly higher than those in S1, S2, S4–S8, S11–S15, S17, and S18 (*p* < 0.01 or *p* < 0.05), indicating that the qualities of FDR decoction pieces prepared using FDRs from Yunnan and Guizhou provinces are significantly better than those prepared using FDRs from 6 other provinces. The contents of components in *F*. *dibotrys* are different when the growing locations are different [13, 22]. *F*. *dibotrys* growing in the high-altitude area of the Yunnan–Guizhou Plateau accumulates more secondary metabolites such as flavonoids and tannins than those growing in the low altitude area, which may be due to the protection of plants from strong ultraviolet radiation and other environmental stresses [2, 23].

SPSS 25 statistical software was used to perform bivariate correlation analyses between the content of one component and the contents of the other 9 components in 23 batches of FDR decoction pieces one by one. The results, presented in Tables 6 and 7, indicate that the contents of procyanidin B3 and catechin exhibit strong correlations with the contents of the other 9 components (*p* < 0.01 or *p* < 0.05), respectively. This finding suggests that only the content of procyanidin B3 or catechin can be detected to estimate the contents of the other 9 components in the future. This approach simplifies the detection process and also saves reference substances.

## 4. Conclusion

In order to provide a reference for rational drug use in clinical practice, this study evaluates the quality of 23 batches of FDR decoction pieces prepared using FDR from Yunnan, Guizhou, Sichuan, Jiangsu, Anhui, Hubei, Henan, and Shaanxi provinces in China by using the method of HPLC fingerprint, Q-TOF-MS/MS and chemical pattern recognition (HCA, PCA, and OPLS-DA) qualitative analysis combined with 10 components (4 tannins such as procyanidin B1, procyanidin B3, procyanidin C1, and procyanidin A2, 3 phenolics such as gallic acid, protocatechuic acid, and protocatechualdehyde, and 3 flavonoids such as catechin, epicatechin, and epicatechin gallate) quantitative analysis. The results show that there are 47 common peaks in the HPLC fingerprints of 23 samples, and 80 components are identified in these 47 common peaks, including 32 tannins, 17 phenolics, 12 flavonoids, 11 PGs, 3 amino acids, 2 organic acids, 1 terpenoid, 1 alkaloid, and 1 other component. FDR decoction pieces prepared using FDRs from Yunnan and Guizhou provinces can be distinguished clearly from those prepared using FDRs from 6 other provinces by HCA, PCA, and OPLS-DA. Except for procyanidin B1 and epicatechin, the average contents of the other 8 components in the decoction pieces prepared using FDRs from Yunnan and Guizhou provinces are significantly higher than those prepared using FDRs from 6 other provinces (*p* < 0.01 or *p* < 0.05). In summary, the qualities of the decoction pieces prepared using FDRs from Yunnan and Guizhou provinces are significantly better than those prepared using FDRs from 6 other provinces.

## Figures and Tables

**Table 1:** Information on samples.

Sample no.	Manufacturers	Batch no.	Habitat of FDR
S1	Anhui Daoyuantang Herbal Pieces Co., Ltd.	211001	Anhui
S2	Beijing Minghui Hengtong Pharmaceutical Co., Ltd.	21121719	Jiangsu
S3	Hebei Hehuachi Pharmaceutical Co., Ltd.	C3082112002	Yunnan
S4	Anhui Wansheng Herbal Pieces Co., Ltd.	211001	Shaanxi
S5	Shanghai Wanshicheng Pharmaceutical Co., Ltd.	20220217-1	Sichuan
S6	Shanghai Wanshicheng Pharmaceutical Co., Ltd.	20211007-1	Sichuan
S7	Shanghai Wanshicheng Pharmaceutical Co., Ltd.	20220304-1	Sichuan
S8	Jiangsu Xinxin Herbal Pieces Co., Ltd.	221117	Anhui
S9	Anguo Tongyi Herbal Pieces Co., Ltd.	20100161	Yunnan
S10	Bozhou Wanzhen Herbal Pieces Co., Ltd.	2106028	Guizhou
S11	Anhui Shenghaitang Herbal Pieces Co., Ltd.	2021051395	Hubei
S12	Sichuan Shengshi Jinrong Pharmaceutical Co., Ltd.	220201	Sichuan
S13	Shandong Baiweitang Herbal Pieces Co., Ltd.	200601	Anhui
S14	Nantong Sanyue Herbal Pieces Co., Ltd.	230308	Anhui
S15	Nantong Sanyue Herbal Pieces Co., Ltd.	230503	Anhui
S16	Anhui Xiehecheng Herbal Pieces Co., Ltd.	22032409	Guizhou
S17	Jiangsu Xinxin Herbal Pieces Co., Ltd.	230206	Henan
S18	Shanghai Kangqiao Herbal Pieces Co., Ltd.	210831	Jiangsu
S19	Anhui Jiayou Herbal Pieces Co., Ltd.	20220401-01	Guizhou
S20	Qujing Gelikang Biotechnology Development Co., Ltd.	20191228	Yunnan
S21	Qujing Gelikang Biotechnology Development Co., Ltd.	20201209	Yunnan
S22	Yunnan Zongshun Biotechnology Co., Ltd.	C200208001	Yunnan
S23	Qujing Gelikang Biotechnology Development Co., Ltd.	20181210	Yunnan

**Table 2:** Components of the common peaks identified by Q-TOF-MS/MS.

Peak no.	*t*_*R*_ (min)	Formula	MS			MS/MS^d^	Identification	Types of compounds	References
			Measured	Theoretical	Error (ppm)				
1	5.386	C_5_H_7_NO_3_	128.0356^a^	128.0353	2.2	128.0344, 82.0296	L-pyroglutamic acid	Amino acids	[13, 16]
2	8.848	C_7_H_6_O_5_	169.0147^a^	169.0142	2.7	125.0243, 169.0159, 97.0296, 107.0149	Gallic acid^e^	Phenolics	[6, 7, 13, 16–18]
3	13.417	C_12_H_14_O_9_	301.0583^a^	301.0565	6.0	169.0140, 125.0244, 301.0571	Pyrogallol-2-O-glucuronide	Phenolics	PubChem
4a	16.616	C_11_H_12_N_2_O_2_	239.0603^b^	239.0593	4.3	116.0518, 74.0277, 203.0880	Tryptophan	Amino acids	[7, 10, 13]
4b	16.749	C_13_H_16_O_10_	331.0681^a^	331.0671	6.7	169.015	Glucogallin	Phenolics	[16]
5	17.291	C_7_H_6_O_4_	153.0198^a^	153.0193	3.1	109.0298, 91.0193, 153.0185, 81.0356	Protocatechuic acid^e^	Phenolics	[6, 7, 13, 16–18]
6	18.416	C_14_H_18_O_9_	365.0664^b^	365.0645	5.2	167.0349, 329.0897, 123.0453	Vanillic acid-4-O-glucoside	Phenolics	[10]
7	21.104	C_13_H_16_O_9_	315.0741^a^	315.0722	6.2	315.0738, 153.0189	Protocatechuic acid 4-O-glucoside	Phenolics	[16]
8	27.035	C_7_H_6_O_3_	137.0249^a^	137.0244	3.8	137.0246, 81.0348	Protocatechualdehyde^e^	Phenolics	[6, 13]
9	28.638	C_15_H_14_O_6_	289.0729^a^	289.0718	3.9	243.1383, 153.0182	(−)-Catechin	Flavonoids	PubChem
10a	31.076	C_19_H_26_O_13_	461.1344^a^	461.1301	9.4	461.1371, 109.0291, 153.0206	Sibiricose A3	Phenolics	PubChem
10b	31.076	C_45_H_36_O_19_	915.1626^b^	915.1545	8.9	727.1516, 709.1290, 557.0807	Epicatechin-(2β ⟶ 7,4β ⟶ 8)-epicatechin-(4β ⟶ 8)-epigallocatechin (7R)	Tannins	PubChem
11	32.369	C_30_H_26_O_12_	577.1435^a^	577.1352	14.4	289.0754, 407.0840, 577.1492, 425.0247	Procyanidin B1^e^	Tannins	[10, 11, 13, 16]
12	32.867	C_30_H_26_O_12_	577.1430^a^	577.1352	13.6	407.0830, 289.0743, 425.0939, 577.1456	Procyanidin B3^e^	Tannins	[10, 16]
13a	33.985	C_45_H_36_O_19_	879.1901^a^	879.1778	14.0	727.1476, 709.1257, 289.0740, 577.1100	Epicatechin-(2β ⟶ 7,4β ⟶ 8)-epicatechin-(4β ⟶ 8)-epigallocatechin (7S)	Tannins	PubChem
13b	34.023	C_15_H_18_O_9_	341.0902^a^	341.0878	7.0	179.0363, 135.0443, 341.0843	1-O-caffeoyl-β-D-glucose	Phenolics	[10]
14	34.715	C_15_H_14_O_6_	325.0514^b^	325.0484	9.1	289.0570, 245.0846, 203.0724, 125.0238	Catechin^e^	Flavonoids	[7, 11, 13, 16–19]
15a	35.136	C_20_H_20_O_14_	483.0834^a^	483.078	11.1	483.0868, 271.0488, 169.0146, 313.0588	1,6-Digalloyl-β-D-glucose	Phenolics	[10]
15b	35.282	C_21_H_24_O_11_	487.1070^b^	487.1013	11.8	331.0869, 451.1322, 269.0849, 289.0734	(+)-Catechin 3-O-glucose	Flavonoids	PubChem
16a	36.832	C_45_H_38_O_18_	865.2118^a^	865.1985	15.3	287.0567, 577.1342, 125.0245	Arecatannin A1	Tannins	[7, 16]
16b	37.039	C_30_H_26_O_11_	561.1471^a^	561.1402	12.2	289.0736, 561.1417, 271.0639, 245.0820	Epicatechin-(3′-*O*-7″)-epiafzelechin	Tannins	[16]
17a	37.788	C_30_H_26_O_12_	577.1426^a^	577.1352	12.9	289.0742, 407.0824, 425.0942, 577.1454	Procyanidin B4	Tannins	[7, 10, 13, 16]
17b	38.381	C_60_H_50_O_24_	1153.2740^a^	1153.2619	10.5	577.1451, 289.0724, 575.1235, 1153.2874	[Catechin-(4α ⟶ 8)]_3_-catechin (4*S*)	Tannins	[7]
17c	38.570	C_17_H_24_O_10_	387.1319^a^	387.1297	5.8	207.0682, 119.0338, 101.0250, 387.1597	Geniposide	Terpenoids	[10]
18a	39.890	C_45_H_38_O_18_	865.2102^a^	865.1985	13.5	287.0584, 451.1077, 713.1683, 865.2332	Procyanidin C2^e^	Tannins	[7, 10, 16]
18b	40.009	C_30_H_26_O_12_	577.1427^a^	577.1352	13.1	407.0832, 289.0751, 425.0943, 577.1453	Procyanidin B2^e^	Tannins	[6, 7, 11, 13, 17]
19a	41.188	C_14_H_22_O_10_	349.1173^a^	349.1140	9.4	99.0439, 249.0623, 349.1152, 113.0253	4,15-Dioxo-5,8,11,14-tetraoxaoctadecane-1,18-dioic acid	Organic acids	PubChem
19b	41.454	C_45_H_38_O_18_	865.2103^a^	865.1985	13.6	287.0575, 577.1424	Arecatannin C1	Tannins	[7, 16]
20a	42.273	C_60_H_50_O_24_	576.1344^c^	576.1273	12.3	1153.2969, 865.1093, 575.1213, 577.1399	[Catechin-(4α ⟶ 8)]3-catechin (4*R*)	Tannins	[7]
20b	42.673	C_45_H_38_O_17_	849.2176^a^	849.2036	15.9	289.0783, 125.0233	Epiafzelechin-(4β ⟶ 8)-epicatechin-(4β ⟶ 8)-catechin	Tannins	PubChem
21a	43.162	C_30_H_26_O_12_	577.1422^a^	577.1352	12.1	577.1502, 289.0789, 407.0790, 425.0927	Proanthocyanidin B2	Tannins	[7]
21b	43.272	C_60_H_50_O_24_	1153.2797^a^	1153.2619	15.4	575.1379, 865.2217	[Epicatechin-(4β ⟶ 8)]_3_-catechin (4R)	Tannins	[7]
21c	43.514	C_52_H_42_O_22_	1017.2252^a^	1017.2095	15.4	559.1281, 575.1515, 729.1502	[Epicatechin-(4β ⟶ 8)]_2_-epicatechin-3-O-gallate	Tannins	[7]
22	44.567	C_15_H_14_O_6_	289.0740^a^	289.0718	7.7	289.0752, 245.0838, 123.0449, 109.0297	Epicatechin^e^	Flavonoids	[6, 7, 13, 16–19]
23	47.388	C_45_H_38_O_17_	849.2164^a^	849.2036	15.0	559.1421, 289.0674, 577.1497, 451.1115	[Epicatechin-(4β ⟶ 8)]_2_-epigallocatechin	Tannins	PubChem
24a	49.628	C_45_H_38_O_17_	849.2154^a^	849.2036	13.9	289.0747, 559.1415, 271.0658, 287.0502	Epiafzelechin-(4β ⟶ 8)-epicatechin-(4β ⟶ 8)-epicatechin	Tannins	PubChem
24b	49.628	C_9_H_8_O_3_	163.0408^a^	163.0401	4.5	119.0507, 93.0350, 65.0378, 163.0385	P-coumaric acid	Phenolics	[13, 16]
24c	49.628	C_17_H_22_O_10_	385.1176^a^	385.114	9.3	267.0741, 385.1174	4-O-sinapoyl-β-D-glucose	Phenolics	[10]
25	51.654	C_45_H_38_O_18_	901.1892^b^	901.1752	15.5	865.2193, 287.0572, 577.1452	Procyanidin C1^e^	Tannins	[7, 10, 13, 16]
26a	53.664	C_16_H_20_O_10_	371.1021^a^	371.0984	10.0	121.0294, 249.0634, 371.1023	Trihydroxycinnamoylquinic acid	Phenolics	PubChem
26b	54.329	C_90_H_74_O_36_	864.2036^c^	864.1907	14.9	865.2198, 125.0229, 289.0752, 577.1421	Cinnamtannin A4	Tannins	PubChem
26c	54.378	C_75_H_62_O_30_	720.1690^c^	720.1590	13.9	289.0723, 125.0247, 287.0568, 577.1461	Cinnamtannin A3	Tannins	[7]
27a	55.796	C_60_H_50_O_24_	576.1360^c^	576.1273	15.1	289.0743, 125.0245, 151.0402, 407.0817	Cinnamtannin A2	Tannins	[7]
27b	55.809	C_11_H_13_NO_3_	206.0832^a^	206.0823	4.5	164.0712, 147.0206, 206.0877	N-acetyl-L-phenylalanine	Amino acids	[10]
28a	58.158	C_45_H_38_O_17_	849.2150^a^	849.2036	13.4	287.0631, 289.0744, 125.0227, 451.1136	Afzelechin-(4 ⟶ 8)-catechin-(4 ⟶ 8)-catechin	Tannins	PubChem
28b	58.158	C_45_H_38_O_18_	901.1873^a^	901.1752	13.4	287.0581, 575.1302, 865.2002, 125.0243	Procyanidin trimer EEC	Tannins	[7]
29a	58.888	C_24_H_28_O_14_	539.1491^a^	539.1406	15.7	539.1544, 197.0548, 327.0763, 211.0617	1,6-Di-O-syringoyl-β-D-glucose	Phenolics	PubChem
29b	59.378	C_75_H_62_O_30_	720.1707^c^	720.1590	16.2	289.0732, 125.0229, 287.0580, 577.1444	Arecatannin A3	Tannins	[7]
29c	59.643	C_18_H_30_O_9_	389.1850^a^	389.1817	8.5	389.1860, 227.1314	Pestalotin 4′-*O*-methyl-β-mannopyranoside	Others	PubChem
30	60.602	C_20_H_20_O_13_	467.0894^a^	467.0831	13.5	467.0908, 423.0989, 315.0749, 313.0854	Ginnalin A	Phenolics	PubChem
31a	62.959	C_21_H_22_O_12_	465.1097^a^	465.1039	12.6	465.1120, 137.0215, 271.1003	Taxifolin-3-glucoside	Flavonoids	[10]
31b	63.008	C_26_H_34_O_11_	557.1873^b^	557.1795	14.0	359.1606	Isolariciresinol 9′-*O*-glucoside	Phenolics	[10]
32a	64.025	C_21_H_24_O_11_	451.1290^a^	451.1246	9.8	303.0747, 141.0197, 451.1318	Epicatechin 5-glucoside	Flavonoids	[10]
32b	64.025	C_15_H_12_O_7_	303.0513^a^	303.0510	0.9	285.0450, 177.0185, 125.0277, 303.0807	Taxifolin	Flavonoids	[10, 13]
33a	65.523	C_14_H_6_O_8_	301.1006^a^	300.9900	5.3	301.01	Ellagic acid	Phenolics	[10, 13]
33b	65.572	C_44_H_34_O_20_	440.0803^c^	440.0749	12.3	125.0230, 169.0141, 303.0533, 407.0813	3,3′-Digalloylprocyanidin B2	Tannins	[7]
33c	65.999	C_59_H_46_O_26_	584.1148^c^	584.1066	14.1	169.0142, 125.0235, 289.0746, 303.0519	Arecatannin A1-3,3′-digallate	Tannins	PubChem
34	66.948	C_22_H_18_O_10_	441.0873^a^	441.0827	10.4	169.0141, 289.0740, 125.0237, 441.0909	Epicatechin gallate^e^	Flavonoids	[13, 18, 19]
35a	67.520	C_27_H_30_O_16_	609.1552^a^	609.1461	14.9	609.1572, 301.0371	Rutin^e^	Flavonoids	[6, 7, 11, 13, 17, 19]
35b	67.557	C_60_H_50_O_24_	1153.2802^a^	1153.2619	15.8	577.1465	Isocinnamtannin A2	Tannins	[7]
35c	67.557	C_45_H_38_O_18_	865.2107^a^	865.1985	14.1	575.1300, 287.0576, 125.0234, 577.1517	Catechin-(4β ⟶ 8)-catechin-(4β ⟶ 8)-epicatechin	Tannins	[7]
36	70.330	C_30_H_24_O_12_	575.2282^a^	575.1195	15.1	575.1294, 289.0749, 423.0809	Procyanidin A2^e^	Tannins	[16]
37	71.570	C_59_H_46_O_26_	584.1159^c^	584.1066	15.9	169.0137, 125.0234, 303.0535, 289.0731	Procyanidin C1-3′,3″-di-*O*-gallate	Tannins	PubChem
38a	80.046	C_33_H_36_O_16_	687.2063^a^	687.1931	19.3	687.2107, 643.2211, 313.0603, 169.0138	Tricin 4′-*O*-(erythro-β-guaiacylglyceryl) ether 7-O-glucoside	Flavonoids	PubChem
38b	80.186	C_31_H_36_O_16_	663.2037^a^	663.1931	16.0	663.2078, 145.0286, 517.1612, 499.1546	1-p-coumaroyl-6-feruloyl-sucrose	PGs	PubChem
39a	80.466	C_37_H_30_O_16_	765.1367^b^	765.1228	18.2	407.0777, 729.1644, 289.0814, 125.0211	Procyanidin B4-3′-*O*-gallate	Tannins	[7]
39b	80.580	C_20_H_20_O_11_	435.0970^a^	435.0933	8.8	435.1055, 109.0303, 315.0730, 137.0214	Taxifolin 3-O-β-D-xyloside	Flavonoids	[10]
40a	84.128	C_32_H_36_O_16_	711.1810^b^	711.1697	15.8	675.2143, 145.0269, 529.1756	6-Acetyl-3′,6′-di-p-coumaroylsucrose	PGs	PubChem
40b	84.352	C_18_H_19_NO_4_	312.1279^a^	312.1241	12.1	148.0533, 312.1300	N-Trans-feruloyltramine	Alkaloids	[10, 13]
41	91.292	C_42_H_44_O_19_	851.2569^a^	851.2404	19.4	145.0288, 851.2882, 663.2099, 705.2299	Tatariside D	PGs	[10, 13, 16]
42	92.803	C_36_H_40_O_18_	759.2309^a^	795.185	22.0	759.2427, 145.0295, 571.1876, 717.2229	Isotatariside A	PGs	[10, 13, 16]
43	94.751	C_44_H_46_O_20_	893.2716^a^	893.2510	20.5	145.0304, 747.2407	Smilaside F	PGs	PubChem
44a	95.452	C_49_H_48_O_20_	955.2824^a^	955.2666	16.5	145.0297, 893.2635	Diboside A	PGs	[10, 16]
44b	95.574	C_18_H_16_O_8_	359.0800^a^	359.0772	7.7	329.0376	5,7,3′-Trihydroxy-6,4′,5′-trimethoxyflavone	Flavonoids	[13]
45a	95.781	C_44_H_46_O_20_	929.2478^a^	929.2276	21.7	894.2553, 145.0258	Lapathoside A	PGs	[10, 13, 16]
45b	95.952	C_50_H_50_O_21_	1021.2759^a^	1021.2539	21.6	145.0287	Lapathoside A	PGs	[10, 16]
45c	96.123	C_51_H_52_O_22_	1051.2856^b^	1051.2644	20.1	145.0288	Lapathoside B	PGs	[16]
46a	97.131	C_18_H_34_O_5_	365.2133^b^	365.2100	9.0	329.2381, 171.1019, 201.1144	9,12,13-Trihyroxy-11-octadecadienoic acid	Organic acids	[16]
46b	97.191	C_40_H_46_O_21_	897.2424^b^	897.2226	22.1	175.0411	1,2′,4′,6′-Tetra-acetyl-3,6-di-feructosyl-sucrose	PGs	PubChem
47	99.090	C_46_H_48_O_21_	935.2790^a^	935.2615	18.7	145.0290	Tatariside C	PGs	[10, 16]

^a^Quasimolecular ion was [M − H]^−^.

^b^Quasimolecular ion was [M + Cl]^−^.

^c^Quasimolecular ion was 1/2[M − 2H]^2−^.

^d^Sequencing according to the abundance.

^e^Confirmed by compared with reference substances.

**Table 3:** Values of the linear relationship, LOD, and LOQ for 10 components.

Reference substance	Regression equation	*R* ^2^	Linear range (ng)	LOD (ng)	LOQ (ng)
Gallic acid	*Y*=2443241*X* − 3282	0.9998	61.215–3060.75	1.705	4.639
Protocatechuic acid	*Y*=1424656*X* − 15956	0.9998	51.765–2588.25	6.977	14.544
Protocatechualdehyde	*Y*=4179072*X* − 18719	0.9999	7.515–375.75	2.262	5.800
Procyanidin B1	*Y*=635497*X* − 50972	0.9998	180.24–9012	31.349	42.114
Procyanidin B3	*Y*=670946*X* − 80886	0.9998	189.36–9468	47.131	63.338
Catechin	*Y*=640535*X* − 23878	0.9998	166.488–8324.4	17.697	29.999
Epicatechin	*Y*=661931*X* − 26122	0.9999	238.368–11918.4	20.842	38.780
Procyanidin C1	*Y*=631157*X* − 267402	0.9999	628.2–31410	151.832	176.587
Epicatechin gallate	*Y*=1056953*X* − 90574	0.9998	256.752–12837.6	32.339	45.318
Procyanidin A2	*Y*=468837*X* − 124600	0.9998	370.8–18,540	97.610	135.308

**Table 4:** Detected values of precision, stability, repeatability, and recovery tests (*n* = 6).

Components	Precision RSD (%)		Stability RSD (%)	Repeatability RSD (%)	Recovery	
	Intraday	Interday			Mean (%)	RSD (%)
Gallic acid	1.02	1.29	1.85	2.48	98.58	1.48
Protocatechuic acid	1.75	1.61	1.20	2.23	100.26	1.22
Protocatechualdehyde	2.32	2.22	2.95	2.70	101.76	1.75
Procyanidin B1	1.67	1.27	2.67	3.15	100.56	0.75
Procyanidin B3	2.12	3.69	1.95	3.43	97.36	1.44
Catechin	1.33	0.98	1.64	1.10	101.13	1.77
Epicatechin	1.13	1.06	2.29	2.00	99.52	1.29
Procyanidin C1	1.11	1.12	3.98	3.56	98.42	1.89
Epicatechin gallate	0.85	0.97	3.20	3.50	99.17	1.23
Procyanidin A2	1.46	2.13	3.22	2.74	98.76	1.25

**Table 5:** The contents of 10 components in 23 batches of FDR decoction pieces (mg/g).

No.	Gallic acid	Protocatechuic acid	Protocatechualdehyde	Procyanidin B1	Procyanidin B3	Catechin	Epicatechin	Procyanidin C1	Epicatechin gallate	Procyanidin A2
S1	0.061	0.094	0.016	0.136	0.183	0.189	0.231	0.537	0.136	0.170
S2	0.012	0.056	0.010	0.040	0.080	0.100	0.211	0.201	0.046	0.100
S4	0.020	0.064	0.010	0.064	0.095	0.066	0.223	0.484	0.240	0.162
S5	0.041	0.076	0.014	0.116	0.144	0.161	0.409	0.849	0.142	0.163
S6	0.042	0.095	0.016	0.139	0.191	0.187	0.447	0.843	0.254	0.215
S7	0.040	0.069	0.012	0.104	0.127	0.125	0.316	0.754	0.098	0.176
S8	0.020	0.025	0.007	0.064	0.067	0.068	0.309	0.576	0.194	0.172
S11	0.048	0.052	0.009	0.110	0.159	0.167	0.456	1.101	0.409	0.204
S12	0.017	0.044	0.008	0.159	0.166	0.155	0.459	0.867	0.420	0.256
S13	0.077	0.076	0.015	0.099	0.147	0.149	0.478	0.925	0.305	0.224
S14	0.059	0.040	0.011	0.140	0.178	0.162	0.947	1.190	0.194	0.161
S15	0.015	0.039	0.009	0.140	0.100	0.167	0.945	1.244	0.207	0.235
S17	0.019	0.024	0.006	0.061	0.064	0.064	0.293	0.549	0.185	0.167
S18	0.018	0.082	0.015	0.053	0.109	0.146	0.325	0.264	0.062	0.121
Average	0.035	0.060	0.011	0.102	0.129	0.136	0.432	0.742	0.207	0.180

S3	0.153	0.079	0.015	0.135	0.179	0.237	0.547	1.113	0.714	0.248
S9	0.161	0.083	0.016	0.142	0.188	0.249	0.577	1.170	0.752	0.271
S10	0.095	0.083	0.015	0.147	0.197	0.191	0.392	1.067	0.688	0.258
S16	0.135	0.044	0.013	0.113	0.151	0.140	0.588	1.077	0.801	0.318
S19	0.118	0.068	0.015	0.165	0.224	0.249	1.240	2.141	0.893	0.362
S20	0.148	0.114	0.023	0.105	0.168	0.178	0.364	1.520	0.408	0.259
S21	0.148	0.110	0.018	0.122	0.184	0.190	0.527	1.410	0.490	0.277
S22	0.230	0.101	0.018	0.106	0.148	0.254	0.495	1.963	0.745	0.212
S23	0.133	0.103	0.021	0.096	0.153	0.160	0.554	1.374	0.510	0.318
Average	0.147	0.087	0.017	0.126	0.177	0.206	0.587	1.426	0.667	0.280

**Table 6:** Results of bivariate correlation analyses between the content of procyanidin B3 and the contents of 9 other components in 23 batches of FDR decoction pieces.

Components	Correlation coefficient of Spearson	*p*
Gallic acid	0.555	0.006
Protocatechuic acid	0.544	0.004
Protocatechualdehyde	0.584	0.003
Procyanidin B1	0.834	0.001
Catechin	0.820	0.001
Epicatechin	0.462	0.027
Procyanidin C1	0.614	0.002
Epicatechin gallate	0.614	0.002
Procyanidin A2	0.629	0.001

**Table 7:** Results of bivariate correlation analyses between the content of catechin and the contents of 9 other components in 23 batches of FDR decoction pieces.

Components	Correlation coefficient of Spearson	*p*
Gallic acid	0.575	0.004
Protocatechuic acid	0.611	0.002
Protocatechualdehyde	0.725	0.001
Procyanidin B1	0.820	0.001
Procyanidin B3	0.509	0.013
Epicatechin	0.722	0.001
Procyanidin C1	0.674	0.001
Epicatechin gallate	0.547	0.007
Procyanidin A2	0.629	0.001

## Data Availability

The data used to support the findings of this study are included within the article.
